# The Central N. Y. Homœopathic Medical Society

**Published:** 1883-08

**Authors:** C. P. Jennings

**Affiliations:** Secretary; Syracuse, N. Y.


### Homœopathic Physician.

A MONTHLY JOURNAL OF MEDICAL SCIENCE.

If our school ever gives up the strict inductive method of Hahnemann, we are lost, and deserve only to be mentioned as a caricature, in the history of medicine."—CONSTANTINE HERING.

Vol. III. AUGUST, 1883. No. 8.

### THE CENTRAL NEW YORK HOMŒOPATHIC MEDICAL SOCIETY.

SYRACUSE, N. Y., March 15th, A. D. 1883, 10 A. M.

The Central New York Homœopathic Medical Society held its quarterly meeting in the office of Wm. A. Hawley, M. D., C. W. Boyce, M. D., in the chair. Sixteen members were present. Chas. T. Harris, M. D., of Syracuse, was elected to membership.

Dr. Hussey submitted a paper on Neural Analysis. Drs. Hawley, Brewster, and Jennings were appointed a committee to consider the practicability of publishing said paper, and to report at next meeting.

Dr. Clausen, from Committee to prepare a protest against the resolution of the Institute, at its last meeting, on freedom of medical opinion, submitted a report.*

Dr. Gwynn—Some time ago we acted upon the subject of consultations with non-homœopathists, and our action was, unfortunately, misunderstood. It would be well for us to be slow in this matter. I move to defer action until our next meeting.

Dr. Nash—I sympathize with Dr. G. in care as to official action. And yet, time enough has passed for us to make up our minds. It

*See pp. 184 and 185 of this Journal for June, 1883.

is important for us to take action before the A. I. H. meets. We do not meet again till June. The A. I. H. meets in the same month. Let Dr. G. have till this afternoon. It is important that we put ourselves on record at this meeting.

Dr. Hawley—The Committee has tried to express pointedly the homœopathic position, and as pointedly our regret at the action of the A. I. H. Its action authorizes a man to resort to any use of drugs he pleases. There is no freedom in homœopathic medicine except in obeying the homœopathic law.

Dr. Brewster—Do we believe in the resolution reported by our Committee? If we do, why hesitate to adopt it? We are men. We have been long studying and practicing Homœopathy. We have come to conclusions as to the principles of the system. If we are at liberty to express our opinion, the sooner we put ourselves on record the better, doing it in a calm and dignified way. We occupy a responsible position. Fidelity to principle and law is the ground on which we stand. The action of the A. I. H. is similar to that of the N. Y. State Society, in dropping the term homœopathic from the the name of our State organization.

Dr. L. B. Wells—It is necessary to act, and to act promptly.

Dr. Clausen—It should be published in all the homœopathic journals.

Dr. Gwynn—After the comments upon what I said, I must add something. I ask Dr. Hawley, Do you not practice with entire freedom of opinion?

Dr. Hawley—Yes; obedience to the law is the liberty I want and try to use. Freedom to go contrary to the homœopathic law is license, not liberty. I do not want liberty to disobey the law and still call myself a homœopathist. That were an absurdity. The point of the resolution of the A. I. H. is that it is allowable for homœopathists to disobey the homœopathic law.

Dr. Gwynn—The resolution of the A. I. H. does not say a word about disobedience to the homœopathic law. Why condemn it? It is nonsense so far as the face of it is regarded. We are imposed upon often by false homœopathists, but we cannot say anything against the resolution.

Dr. Clausen—Societies are formed for usefulness, development, and progress. To accomplish these successfully, some essential tenets must be the rule. These tenets involve three propositions: First, that which is called a science can be a science only as it is governed by law; second, law, to be law, must be universally applicable to the science it governs; third, any deviation from that law is unscientific and empirical, and can reap only spurious results.

The American Institute of Homœopathy was formed for the promotion of medical science. Its founders recognized for that science a law; they recognized that law to be "*similia similibus curantur.*" They therefore logically recognized Homœopathy as the science of therapeutics—being a science by virtue of having a law. It therefore follows that the promotion of medical science can result only from strict conformity to the *law*. It is only the perfect law of liberty that delivers us from the bondage of lawlessness and recklessness, with their direful punishments. The lawless are always slaves to their own waywardness; they enjoy license, but not the perfect law of liberty. Strenuous efforts have been made by certain giddy-headed communists in our ranks to unite the common school of medicine with ours. We have deplored the inconsistency and the irrationality of this crazy idea. We have read that disgraceful and abominable resolution, which was passed at the last meeting of the Institute in 1882, touching "absolute freedom of medical opinion and unrestricted liberty of action." It was introduced and passed at the close of the meeting, when very few members were present. It is the most absurd of all steps yet taken by the Institute. It is the grand climax of the perversion of Carroll Dunham's plea for liberty. Its advocates delight in old-school recognition; but they cannot be consistently loved by the honest, even in the common school of medicine.

We, who are gathered here to-day, represent the Central New York Homœopathic Medical Society. Our Society was formed in 1866 in the interests of pure Homœopathy. "Through all vicissitudes it has adhered firmly to the fundamental principles of Homœopathy, viz.: The *simillimum*, the single remedy, and the *minimum* dose."

It is our solemn duty, in honor to truth and loyalty, to protest with emphasis against every departure from ever-efficient Homœopathy, and from its unerring law.

The report was accepted and adopted, and is as follows:

Ordered, that this protest be published in all the homœopathic journals of the country.

Reports of provings were called for.

Dr. Emens—Have tried the trituration distributed by the secretary. No results.

Dr. Nash—All of us may take this trituration and have no results, because not susceptible. Persons differ in susceptibility. Moreover, a prover should be very systematic and persevering. We must try such attenuation as will best meet our susceptibility.

Dr. Boyce—Hahnemann says the power of drugs is increased by potentizing. See *Organon*, Par. 128, Stratton's translation. He says we obtain powerful effects from the 30th. Shall we give up trying to prove remedies? Can we prove Hahnemann's statement either false or true? This is the first attempt at proving we have made as a society. Shall we go on with the provings of this drug? We ought to obtain results from the 30th. The Austrian provers sought to disprove Hahnemann's teachings on this subject. But they confessed that they obtained more and better symptoms from *Natrum mur.*$^{30}$ than from lower potencies of the same drug.

Dr. Clausen.—Let us repeat the effort to prove this drug, and agree to take it in solution.

Dr. Schmidd—Would it not be well to try some medicine not yet sufficiently proved? *Syphilinum* cured a case for me having the peculiar symptom of pain coming on at sunset and ending at daybreak. It was the only symptom I followed. One dose cured him perfectly in two weeks. This remedy is not sufficiently proved. So with *Lac caninum*. A symptom is where the pain keeps changing from side to side. We have a number of medicines not proven.

Dr. Boyce—We should study Hahnemann's *Organon* from Par. 105 to 145, and follow his instructions with close exactness when proving drugs.

Ordered, that this proving be continued the next three months.

Dr. Boyce—Dr. Schmidd has spoken of *Syph.* Has any one else had any experience with it?

Dr. Nash—A child had caries of the spine. The occiput sank down, resting on the protuberance of the curvature. There was a swelling. Fluctuation led to opening it. Remedies failed. Saw Dr. Skinner's statement of a cure of caries of the spine with *Syph.* The child complained it pricked, and was worse at night. Nitric acid failed. The matter became almost purely calcareous. Old-school physicians said the child would die. The mother said the child always called any pain a pricking. Gave Swan's *Syph.*$^{cm}$, once in ten days, till three doses were given. Cured the caries. There was no positive proof of syphilis in the case. Exacerbation at night is one indication for this remedy.

Dr. Boyce—I gave it in a case of dirty eruption of the scalp. Cured. No special indication for giving it.

Dr. Hawley—A case of syphilis. Sore throat. Copper-colored maculæ from crown of the head to the soles of the feet. Gave Swan's *Syph.*$^{cm}$, three doses in five months. Complete cure. Sore throat disappeared almost immediately.

Another case: Nose and cheeks covered with eruptions and scabs. Scabs in layers rising to a point. The first dose of *Syph.* took off the scabs in two weeks.

Another case, the wife of the first patient: Tried it on her without effect.

Another case: Inguinal bubo. Remedies failed. *Syph.* cured completely.

Nosodes should be proved in order to obtain indications as to when they are homœopathic.

Dr. Schmidd—Will the high potency annihilate the aggravations of lower potencies? I have found it true. Nosodes should not be given simply to antidote their own specific poison. They should be proved, and given only as indicated.

Dr. Clausen—A patient had night sweats; was sleepless and restless; had suffered previously from an attack of ovaritis. *Syph.*$^{cm}$, three or four doses within two days. Relieved after the second day.

Calcarea carb. was taken up.

Dr. Clausen—Have used it in several cases of too early and profuse menstruation with good results. Regard this as the characteristic indication. There were other symptoms, such as cold and damp hands and feet.

Dr. Schmidd—Find it useful for small children in winter cough. Calcarea constitution; sweat on the head, wetting the pillow; hard stools; cold hands; wheezing breathing; canine appetite; drink all the time; milk comes up curdled; fever at 2 P. M., goes down and rises again at night. Good results from the MM potency. Have had exacerbations in children from high potencies; aggravations produced the second or third day; the exacerbations pass away, leaving the patient cured.

In grown persons Calcarea is indicated by such symptoms as these: The patient is phlegmatic; unhappy; there is pressure on the head; she cannot endure the same; profuse and too early menstruation; Calcarea constitution. Patient should be fleshy and light complexioned. Canine hunger, wants to eat all the time. Have seen intermittent fever cured by it in six doses, where there had been great unhappiness as a prominent symptom.

Dr. Martin—A case in my first year of Homœopathy. A plump, fleshy child had intermittent fever. Gave old-school remedies in vain. The child became very sick; emaciated; complexion sallow; temples a lilac color. Gave Calcarea carb. empirically, not being acquainted with Homœopathy then. The child improved immediately.

Dr. Seward—A child, five months old, black eyes and hair, had cholera infantum; face old, blue, pale. One dose of Calcarea carb.$^{30}$, cured.

Another child was troubled with constipation; would cry and strain sometimes, and have a very large stool. Calcarea carb. cured.

A woman had lung trouble. She could not lie down; feet dropsical, the swelling extending to the knees; taste bad; tongue foul; breath fetid; no appetite. Gave one dose Calcarea carb.$^{30}$. She grew better immediately. The second night she could lie down. Cough cured.

Dr. Young—A boy, about six years old. The mother fleshy and heavy, of an easy, good nature. The boy had intermittent fever. Grew worse. His physicians gave him up to die. In sleep, great drops of sweat would break out on his face; feet and hands cold; he wanted to sleep all the time. Calcarea carb.$^{200}$ cured.

Another boy was attended by an allopathist, who said he used Homœopathy when wanted. Dysentery had run the boy down. Skin dry, and looked like chalk; the sutures of the skull were open. Nux vom. had been given. It made matters worse. I gave Calcarea carb. and cured him. There was marasmus in this case; abdomen prominent.

I give Calcarea carb. when a child's teeth develop badly, and rot as soon as they come out. [*Kreos.*, Staph.—Ed.]

Dr. Nash—In children when development of the bony system is hindered, there should be careful discrimination between Cal. carb., Silicea, and Sepia. Cal. carb. acts most promptly where are the symptoms which have been spoken of, when the vomiting is curdled, and the stools are sour. Where these last symptoms are not present, and the temperament is wiry, Silicea helps; Sepia when the child cannot take boiled milk. Have found Cal. carb. useful in lumbago, and in sprains of the back where Rhus tox. has failed me, and the patient has been working in the water.

Dr. Boyce—Vomiting on waking up in the night led me to give Cal. carb. and it cured the patient.

Dr. Brewster—A cake of milk crust in a nursing babe, fat and flabby. It had been under treatment five to seven months. A cap had formed on the head. On each cheek was a patch covered with a crust of the same kind. Cal. carb.$^{8c}$ removed the cap in two weeks. Several weeks more were occupied in perfecting the cure.

In one case the child had been three months under treatment, and one eye was destroyed. Then the child was put under my care. Cal. carb. cured it, and saved the other eye, which had been involved.

Dr. Schmidd—Generally one dose effects the cure.

Dr. Seward—Cal. carb. is indicated when there is a sensation of coldness of the head, must keep it covered.

Dr. Baldwin—Have had, and have seen, fine results from Cal. carb. in phthisis; expectoration thick, heavy, and yellowish; perspiration sour and sticky, especially at night, making the clothing sticky; the sweat on hands and feet feels cold to the patient. I give the 30th, and repeat till results are obtained.

Dr. Nash—I repeat Cal. carb., as I do every medicine, until there is some effect.

Dr. Schmidd—Have antidoted the morbid effects of repeating lower potencies by one dose of a high potency. But the matter is not settled in my mind.

Dr. Hawley—Have had much success with Cal. carb. A child, six years old, had suffered from epilepsy three or four years. Paroxysm every two weeks. He had been under old-school treatment. Came under my care. He was thin and pale; ears were translucent; feet and hands cold and wet. One dose of Cal. carb.$^{2c}$ was given. He is now a healthy youth of seventeen. Has not had another fit. I use this remedy almost every day for adults and children.

Dr. Brewster—A case of sick headache, vomiting in the night. Cured with Cal. carb.

Dr. Southwick—A case of epilepsy. The disease came on after falling into the water. A peculiar sensation would start at the stomach and spread all over. Took drugs. Went on eighteen years. Cal. carb., once a month, has improved the case greatly.

Dr. Schmidd—One dose Cal. carb.$^{cm}$ cured this symptom: everything looks to be at a distance.

Dr. Hawley—Cal. carb. is called for when the patient is miserable all the forenoon, better about four or five P. M.

Dr. Schmidd was requested to prepare a paper on the proving of drugs.

Dr. Baldwin was appointed a committee to ascertain the cost of printing one hundred copies of the constitution and by-laws.

Dr. Seward read a paper, giving an account of use of Lachesis.

Adjourned.

C. P. JENNINGS, *Secretary.*

